# Testosterone levels among non-obstructive azoospermic patients 2 years after failed bilateral microdissection testicular sperm extraction: a nested case-cohort study

**DOI:** 10.1007/s10815-022-02497-x

**Published:** 2022-04-25

**Authors:** Charles C. Herndon, Erica S. Godart, Paul J. Turek

**Affiliations:** 1 The Turek Clinic, 9033 Wilshire Blvd, Suite 408, Beverly Hills, CA 90211 USA; 2 College of Osteopathic Medicine, Touro University Nevada, Henderson, NV USA

**Keywords:** Azoospermia, Hypogonadism, Microdissection, Testicular sperm retrieval, Testosterone

## Abstract

**Purpose:**

To define the risk of hypogonadism following microdissection testicular sperm extraction in cases of non-obstructive azoospermia. While sperm retrieval by open testicular sperm extraction can be associated with an increased risk of hypogonadism, there is limited data addressing which procedures and which patients harbor the greatest risk.

**Methods:**

We report on a community-acquired, nested, case-cohort of non-obstructive azoospermic patients referred to one clinic after failed bilateral microdissection testicular sperm extraction. Patients were health-matched (1:2) to surgically naïve controls and divided into 2 cohorts based on risk factors for hypogonadism. Among microdissection patients, we compared total testosterone and gonadotropin levels before and > 6 months after surgery. Biochemical hypogonadism was defined as a total serum testosterone level ≤ 300 ng/dL. Hormone levels were compared to risk-matched controls. Comparative statistics were used to assess hormone levels within and between cohorts.

**Results:**

There were no significant differences in baseline testosterone levels between microdissection patients (*n* = 26) and risk-matched controls (*n* = 52). At a mean of 26 months (range 6.2–112.8) post-procedure, mean testosterone levels decreased significantly (73 ng/dL or 16%; CI − 27, − 166; *p* < 0.01, paired *t*-test). Among microdissection patients with baseline testosterone > 300 ng/dL, 8/22 (36%) experienced hypogonadism post-procedure. There was a corresponding increase in follicle stimulating hormone (*p* = 0.05) and a trending increase in luteinizing hormones (*p* = 0.10).

**Conclusion:**

A durable decrease in testosterone levels occurs after failed microdissection testicular sperm extraction regardless of baseline risk of hypogonadism. In addition, a significant proportion of eugonadal patients will become hypogonadal after failed testicular microdissection procedures.

## Introduction

Testicular sperm retrieval is now almost 30 years old [1]. It followed the invention of IVF-ICSI in 1992 and, for the first time, allowed men with azoospermia due to either testis failure or unreconstructable blockages the opportunity to become biological fathers [2]. One of the earliest observations made from experience with testicular sperm retrieval is that it is typically more difficult to find sperm in patients with non-obstructive azoospermia (NOA) than it is in those with obstructive azoospermia (OA). This is largely because there is reduced sperm production in men with NOA, and as a consequence, spermatogenesis can be “patchy,” occurring in “islands,” unlike the uniformly and globally normal sperm production in men with obstruction [3, 4]. Indeed, this has led clinicians to develop several creative strategies to optimize the success of testicular sperm retrieval procedures, including the multi-biopsy testicular sperm extraction (TESE) [5], microdissection TESE (microTESE) [6], and fine-needle aspiration (FNA) mapping followed by map-guided TESE procedures [7]. As to which technique is “best,” three Cochrane reviews of the literature and a very recent, large, systematic meta-analysis note a lack of randomized controlled trials from which to base any recommendation [8–11]. The only hard and fast conclusion was to select the least invasive and simplest technique for sperm retrieval whenever possible [9].

Given the lack of agreement regarding which procedures are best for retrieving testicular sperm in patients with NOA, the issue of risk or “invasiveness” becomes paramount. This is especially true since surgical procedures fail to find sperm in up to 50% of NOA cases [12]. Interestingly, there is scarce literature examining the health consequences of surgical sperm retrieval procedures [13–15]. The risks of testis “devascularization” and “scar” (by ultrasound) and “seminiferous tubule diameter” (by histology) have been used as surrogates of “damage” or invasiveness of sperm retrieval procedures [13–15]. However, these outcomes lack clinical correlates to health. The single most relevant outcome of the invasiveness of a testicular surgical procedure is surgically induced hypogonadism or low testosterone levels, potentially requiring life-long hormone replacement.

There is limited published data on the effects of testicular sperm retrieval procedures on hypogonadism. It is generally believed that the smaller the TESE samples taken, the less chance of postoperative hypogonadism [16]. With a single albeit large incision and more precise tissue dissection, microTESE was initially thought to be less invasive than conventional TESE procedures. However, with time and wider experience, it appears that serum testosterone levels recover to baseline in only 50–90% of patients 1 year after microTESE in experienced hands and with adequate clinical follow-up [17–21]. A novel physiological study examined Leydig cell function after TESE procedures by employing a human chorionic gonadotropin (hCG) stimulation test postoperatively [22]. Among NOA patients with *normal* serum testosterone levels before or after TESE, 40% demonstrated a weaker than normal testosterone response to hCG stimulation after TESE procedures, indicating compromised Leydig cell function. Thus, cohort studies to date suggest that testicular sperm retrieval procedures have an effect on testosterone balance and potentially hypogonadism.

The first systematic review and meta-analysis of 15 non-randomized, retrospective, uncontrolled studies of testosterone levels before and after TESE procedures shed more light on this issue [23]. Among men with OA and NOA having TESE procedures, a statistically significant decrease in testosterone levels occurred for up to 12 months after the procedure that put patients at risk of “temporary hypogonadism.” The degree of impairment was most marked in men with Klinefelter syndrome. A full recovery of mean testosterone levels was noted at 18 months among study cohorts. Significant limitations of this analysis include a wide heterogeneity of procedures performed (i.e., TESA, TESE, and microTESE), the inclusion of OA and NOA patients which likely have different risk profiles for hypogonadism, and that mean cohort testosterone levels were analyzed instead of individual levels which likely underestimates any effect in individuals. In other words, although a cohort of patients may show full recovery of testosterone levels postoperatively, this does not exclude the possibility that a subset of individuals within the cohort does not recover testosterone levels.

Thus, the current literature suggests that there is a significant risk of temporary and even permanent hypogonadism after TESE procedures. What is unclear is which procedures and which patients have the highest risk of hypogonadism. We examined testosterone levels before and after sperm retrieval in eugonadal and hypogonadal NOA patients who underwent microTESE to better understand the risk of hypogonadism after this procedure.

## Materials and methods

### Study design and populations

In this retrospective, nested case-controlled cohort study, the clinical characteristics of two populations of NOA patients were compared. The overall study population consisted of community-acquired NOA patients referred to a single male infertility clinic. One subpopulation was patients who failed microTESE procedures performed elsewhere. Failed microTESE procedures were defined as those in which no sperm was extracted for medically assisted fertilization. The second population (controls) consisted of contemporary NOA patients who did not have prior diagnostic or therapeutic sperm retrieval procedures. This study was conducted with institutional review board approval (Solutions IRB, Protocol #2019/04/15; Yarnell, AZ).

All patients had a detailed history, physical examination, and laboratory testing, including serum total testosterone, follicle-stimulating hormone (FSH), and luteinizing hormone (LH) levels as part of their visit to the clinic. The historical, pre-procedure laboratory characteristics of the microTESE cohort were collected from a review of patients’ past medical records of care received elsewhere. Study inclusion criteria for both NOA cohorts included men with any root cause, the procedural criteria above, and complete clinical and laboratory data available before and after microTESE procedures. Notably, patients with non-mosaic Klinefelter Syndrome (47, XXY) were uniformly excluded from the study due to the high risk of baseline hypogonadism in this population. In addition, the post-microTESE clinical and laboratory evaluation must have been performed > 6 months after the microTESE procedure to allow time for testicular healing and recovery. All patients must have discontinued aromatase inhibitors, chorionic gonadotropin injections, testosterone replacement therapy, and any other hormone therapies at least 3 months prior to surgery and > 6 months post procedure. In cases of serial laboratory values, the most recent measurements were included in the analysis. We sought to generate a cohort of NOA controls that were clinically similar to the microTESE patients for comparison purposes. To this end, we applied case-match criteria to age- and BMI (body mass index)-match the two populations of patients at a 2:1 ratio of NOA controls: microTESE patients.

An analysis was also performed to identify potential NOA patients who might be at higher risk of surgically induced hypogonadism. Based on baseline health and hormone status, both NOA control and microTESE patients were subcategorized as follows: We defined “high-risk” subjects as those with a history of testicular surgery or trauma, chemotherapy, radiotherapy, undescended or solitary testicles, and those presenting with biochemical hypogonadism at baseline (< 300 ng/dL). The “low-risk” group included those without these and other fertility-related comorbidities and the absence of baseline hypogonadism.

### Outcomes

The primary outcome was whether microTESE procedures significantly alter serum reproductive hormones, including testosterone, follicle-stimulating hormone (FSH), and luteinizing hormone (LH). Pre-procedure hormone levels among microTESE patients were compared to NOA controls to evaluate whether the microTESE cohort was clinically comparable to control NOA men. In addition, pre-procedure hormone levels among microTESE patients were compared to post-microTESE levels in this cohort. A secondary outcome was to determine the incidence of surgically induced hypogonadism among microTESE patients. This analysis focused on patients who were eugonadal (serum total testosterone > 300 ng/dL) prior to microTESE and who subsequently became hypogonadal (serum total testosterone < 300 ng/dL) post-procedure [24]. A third outcome assessed whether risk-stratification could allow us to identify individuals at the highest risk of surgically induced hypogonadism.

### Statistical analysis

Data was collected and analyzed in Microsoft Excel (Microsoft, Seattle, WA). Baseline hormone levels were evaluated as means and standard deviations and were compared within and between cohorts using the independent sample *t*-test. Post-procedure hormone levels were compared to baseline levels by a paired samples *t*-test. In addition, the analysis of risk-stratified subjects for surgically induced hypogonadism was assessed with paired sample *t*-tests. Statistical significance was considered as a two-tailed value of *p* < 0.05. The incidence of surgically induced hypogonadism after microTESE procedures was assessed by simple descriptive statistics.

## Results

### Patient selection

**Fig. 1:**
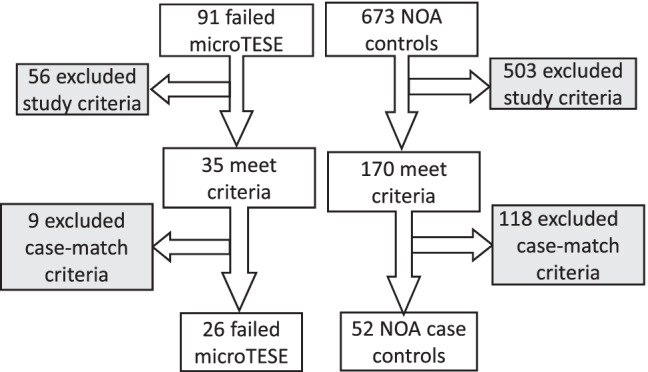
Schematic of patient study flow. Two filters (gray boxes) were applied to populations of non-obstructive azoospermic men. The first filter was general study inclusion criteria, as outlined in the methods. The second filter applied case-match criteria to age- and body mass index match the two populations at a 2:1 ratio of NOA controls: microTESE patients. Note: NOA=non-obstructive azoospermia; microTESE=microdissection TESE

Study subject flow and cohort selection are outlined in Fig. 1. Over a 9-year period (2000–2019), 734 consecutive NOA patients were evaluated at the clinic. Among them, 91 had failed microTESE procedures elsewhere, and 673 had no prior sperm retrieval procedures. After applying the filter of general selection criteria, we obtained 35 microTESE patients and 170 NOA controls. Adding the filter of case-matching for age and BMI, we obtained the final study cohort populations of *n* = 26 microTESE patients and *n* = 52 NOA controls. Nine microTESE patients were excluded from the analysis due to a lack of matched controls with an age ± 5 years and a BMI ± 5 points. A demographic comparison of the two NOA cohorts is outlined in Table 1. There were no statistical differences in age or BMI status between study cohorts.

**Table 1:** Demographic comparison of two NOA cohorts

Patient Demographics		Age^b^	BMI^b^
MicroTESE *n* = 26 patients	Average ± SD	33.3 ± 5.5	28 ± 5.6
	Range	25.3–47.7	22.2–44.2
Control NOA^a^ *n* = 52 patients	Average ± SD	34.2 ± 4.1	26.7 ± 3.7
	Range	26.7–45.7	21.4–37.6

^a^Non-obstructive azoospermia

^b^No statistically significant difference in age or BMI

### Comparison of testosterone levels

**Fig. 2:**
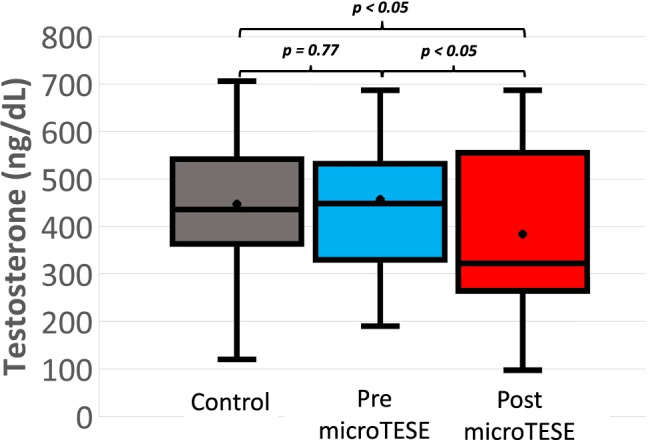
Comparison of testosterone levels between controls and pre- and post-microTESE patients. Independent samples *t*-test found no statistically significant difference between controls and pre-microTESE patients, but there was a significant difference between controls and post-microTESE patients (*p* < 0.05). A paired samples *t*-test comparison between pre- and post- microTESE patients showed a significant difference (*p* < 0.05). NOA=non-obstructive azoospermia; microTESE=microdissection TESE

The mean total testosterone level among NOA controls was 447 ng/dl (SD ± 116; normal range 300–800 ng/dL) and among pre-microTESE patients was 457 ng/dL (SD ± 158). An independent samples *t*-test showed no significant difference (Fig. 2). The mean testosterone level among post-microTESE patients was 384 ng/dL (SD ± 178), which represents a 73 ng/dL mean reduction (16%) in testosterone levels after microTESE procedures. A paired samples *t*-test showed this to be a statistically significant difference (CI − 27, − 166; *p* < 0.01, paired *t*-test) (Fig. 2). The mean interval between microTESE procedures and post-procedure hormone testing was 26.0 months (range: 6.2–112.8 months).

### Comparison of gonadotropin levels

**Fig. 3:**
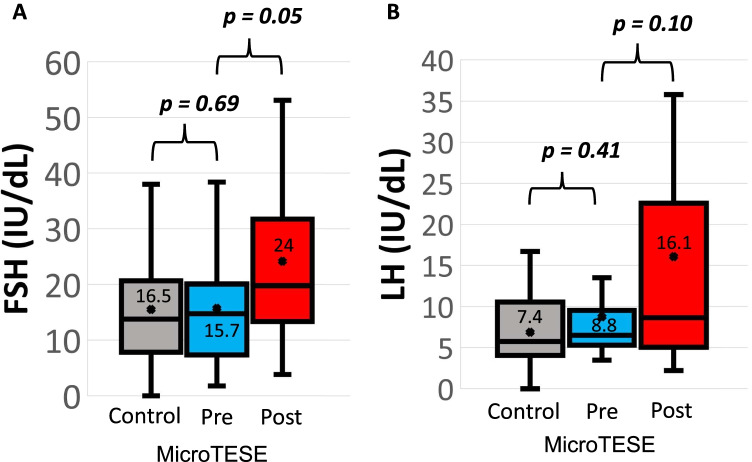
Comparison of mean gonadotropin levels between controls and pre- and post-microTESE patients. FSH (panel **A**) and LH levels (panel **B**) were similar between pre-microTESE patients and NOA controls. However, there was a significant compensatory elevation of FSH levels post-microTESE when compared to pre-microTESE levels. There was a trend toward increased LH levels after microTESE compared to pre-microTESE. microTESE microdissection TESE

Baseline FSH levels were similar between microTESE patients and NOA controls (16.5 IU/dL vs. 15.7 IU/dL, respectively, *p* = 0.69, means *t*-test; normal range 1.5–8.1 IU/dL) (Fig. 3A). Likewise, baseline LH levels were also comparable between microTESE patients and NOA controls (7.4 IU/dL vs. 8.8 IU/dL, respectively, *p* = 0.41, means *t*-test; normal range 1.5–9.3 IU/dL) (Fig. 3B). The significant drop in total testosterone levels after microTESE procedures was associated with a compensatory elevation of gonadotropins (Fig. 3). FSH levels post-microTESE (24 IU/dL) increased significantly when compared to baseline (15.7 IU/dL) by sample means *t*-test (*p* = 0.05). There was a trend of increased LH levels after microTESE (8.8 IU/dL baseline vs. 16.1 IU/dL post-microTESE, respectively), but the difference was not statistically significant (*p* = 0.10, sample means *t*-test).

### Rate of surgically induced hypogonadism

**Fig. 4:**
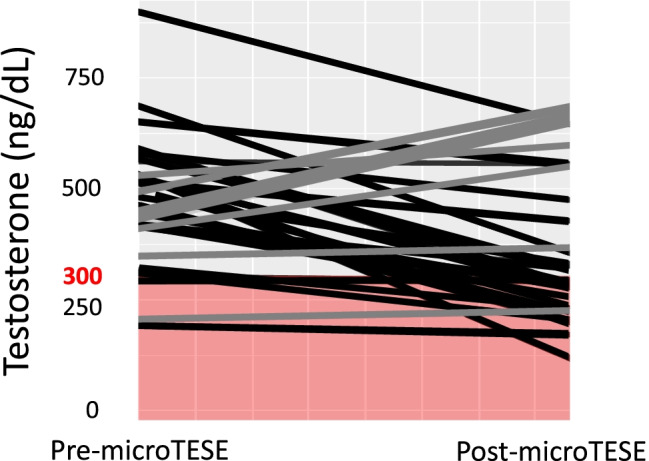
Eugonadal to hypogonadal conversion after microTESE procedures. A comparison of individual testosterone levels among microTESE subjects is illustrated. On the left are the baseline, pre-microTESE testosterone levels, and on the right are the post-microTESE levels. The change in individual testosterone levels is shown by a gray bar (same or increase in levels) or a black bar (decrease in levels) in individual subjects. The red shading in the graph indicates a testosterone level below 300 ng/dl of testosterone, which is considered hypogonadal. Among 22 microTESE patients with normal baseline testosterone levels, 8 (36%) converted to hypogonadism post-microTESE. The mean interval between microTESE procedures and post-procedure hormone testing was 26.0 months. microTESE microdissection TESE

As shown in Fig. 4, among the 22 microTESE patients with normal (> 300 ng/dL) baseline total testosterone levels, 8 (36%) converted to hypogonadism post-microTESE. The mean interval between microTESE procedures and post-procedure hormone testing was 26.0 months (range: 6.2–112.8 months).

### Risk stratification for surgically induced hypogonadism

**Fig. 5:**
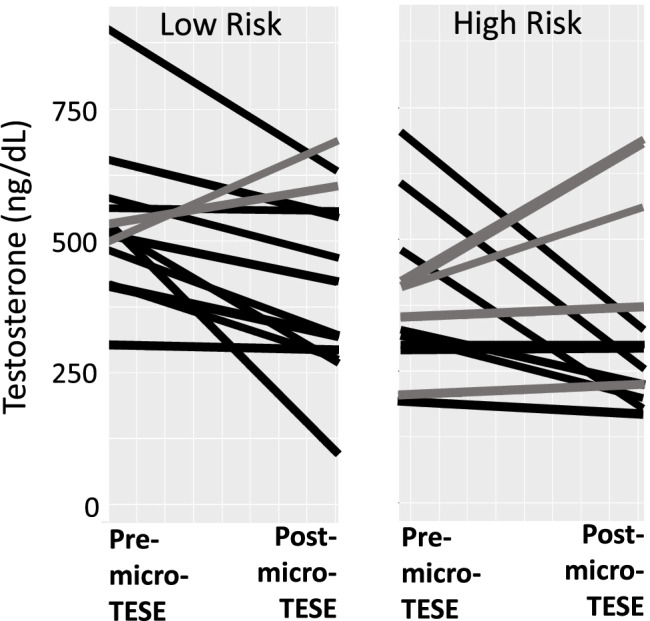
Comparison of pre- and post-microTESE testosterone levels within low-risk and high-risk cohorts. The left graph represents microTESE patients (*n* = 13) designated as having a low risk of post-microTESE hypogonadism. The right graph represents patients (*n* = 13) designated as high risk of hypogonadism. In each graph, baseline, pre-microTESE testosterone levels are on the left and post-microTESE levels are on the right. The change in individual testosterone levels is shown by a gray bar (same or increase in levels) or a black bar (decrease in levels). Notably, the mean percent decrease in testosterone levels after microTESE procedures was 19% and 6% in the low-risk and high-risk cohorts, respectively. microTESE=microdissection TESE

The mean baseline total testosterone levels in the high-risk (*n* = 13) and low-risk (*n* = 13) microTESE subjects were 383 ng/dL and 531 ng/dL, respectively, and were significantly different (*t* test; *p* < 0.01). Comparing baseline and post-microTESE testosterone levels in the low-risk cohort, there was an absolute decrease of 109 ng/dL, which was statistically significant (CI − 12.5, − 203; *p* < 0.05, paired *t*-test). In the high-risk cohort, the mean absolute decrease in testosterone levels was 39 ng/dL following microTESE procedures, which was not statistically significant (*p* = 0.48). Analyzed differently, the mean percent decrease in testosterone levels after microTESE procedures was 19% and 6% in the low-risk and high-risk cohorts, respectively. The change in testosterone levels in the low- and high-risk microTESE cohorts is shown graphically in Fig. 5.

## Discussion

There is a substantial body of literature that addresses the success of testicular sperm extraction (TESE) procedures [25]. However, there is far less information available about the health consequences of surgical sperm retrieval procedures. Although testis devascularization and scar and seminiferous tubule diameter have been assessed, these outcomes lack clinical correlates to health [13–15]. Clearly, the most health-relevant outcome of the invasiveness of a testicular surgical procedure is surgically induced hypogonadism or low testosterone levels that may require life-long hormone replacement. A recent systematic review and meta-analysis of 15 non-randomized, retrospective, uncontrolled studies has been the most informative to date on this issue [23]. It suggested that there was a statistically significant decrease in testosterone levels for up to 12 months after TESE procedures that put patients at risk of temporary hypogonadism. Notably, permanent hypogonadism was not observed when mean cohort testosterone levels were evaluated over 18 months. Unfortunately, this study included all types of TESE procedures and evaluated men with both NOA and obstructive azoospermia, making the study findings less applicable for men with NOA who require the most invasive TESE procedures to find sperm. We examined testosterone levels before and after microTESE procedures in a nested case-cohort analysis of eugonadal and hypogonadal NOA patients to better define the risk of surgically induced hypogonadism.

Our results suggest that there are significant health implications related to failed microTESE procedures in men with NOA. Although baseline testosterone levels were similar among NOA men who did and did not undergo microTESE procedures, there was a significant decrease in testosterone levels after microTESE procedures when examined with 2 years of follow-up. In addition, 36% of patients who were eugonadal at baseline remained hypogonadal after microTESE procedures. Not unexpectedly, these changes in testosterone levels were associated with significantly elevated FSH levels and a trend toward LH elevation, further corroborating the physiological effect of microTESE procedures on testicular function [18]. Lastly, we attempted to define populations of NOA men who are more likely to become hypogonadal after microTESE procedures. We observed decreases in testosterone levels in both high- and low-risk NOA cohorts, but, somewhat unexpectedly, the magnitude of the testosterone decrease was greater in low-risk than high-risk patients. This could reflect the fact the reproductive microsurgeons might be surgically more aggressive with microTESE procedures in eugonadal more than hypogonadal men. Or, it might be a difference that would regress to the mean and lose significance if a much larger study were conducted. Either way, we conclude that our profiling of NOA patients into high- and low-risk categories of hypogonadism is not clinically helpful at this time.

Significant strengths of the study are the nested case-cohort study design and the complete hormonal and clinical follow-up of subjects. Nested case-cohort studies can efficiently evaluate the relationship between an exposure and a single outcome or disease, in this case, hypogonadism. Our control population of NOA men who were health-matched to microTESE patients revealed that the microTESE patients were typical or representative of NOA patients in general. Also, the study examined hormone levels for a mean of over 2 years post microTESE procedures, which is sufficient to allow for complete testicular recovery and healing [23]. A study weakness is the fact that patients were not followed clinically to see if they actually developed symptomatic hypogonadism, which would justify testosterone replacement [24]. Furthermore, this study does not address changes in testosterone levels among patients having successful microTESE procedures. Presumably, successful microTESE procedures would involve less risk of hypogonadism as they are typically terminated as soon as sperm are located. In many such cases, only unilateral procedures are performed and often limited microTESE procedures at that. So, this study of failed microTESE procedures likely represents the “worst-case” scenario among surgical sperm retrieval procedures. In addition, microTESE procedures in this study were performed by a variety of microsurgeons worldwide, all with likely differing surgical skillsets. This fact also makes the results more generalizable to the NOA population at large, as it represents a “real-world” experience and not “efficacy” data from a single center [25]. Real-world findings on microTESE procedures have greater implications for men’s health than do single-center data.

## Conclusions

Take-home observations from this study include the fact that NOA men undergoing failed microTESE procedures are at definite risk of post-procedure hypogonadism. However, we were unable to identify which patients were at the highest risk of hypogonadism. Certainly, the risk of hypogonadism and its associated health consequences should be discussed with patients who are considering microTESE sperm retrievals.
